# Supplementary material for: Altered microRNA Transcriptome in Cultured Human Liver Cells upon Infection with Ebola Virus
Source: Int J Mol Sci. 2021 Apr 6;22(7):3792. doi: 10.3390/ijms22073792 (PMC8038836; doi:10.3390/ijms22073792)
Supplement: Supplementary file 1 [file ijms-22-03792-s001.zip › Supplementary_File/D_ KEGG_Analysis_Results/16-30nt_pathway_Reston-96h-Huh7_vs_Control-96h-Huh7_up.mature_mirna_targets/hsa_EnrichmentScore.pdf]

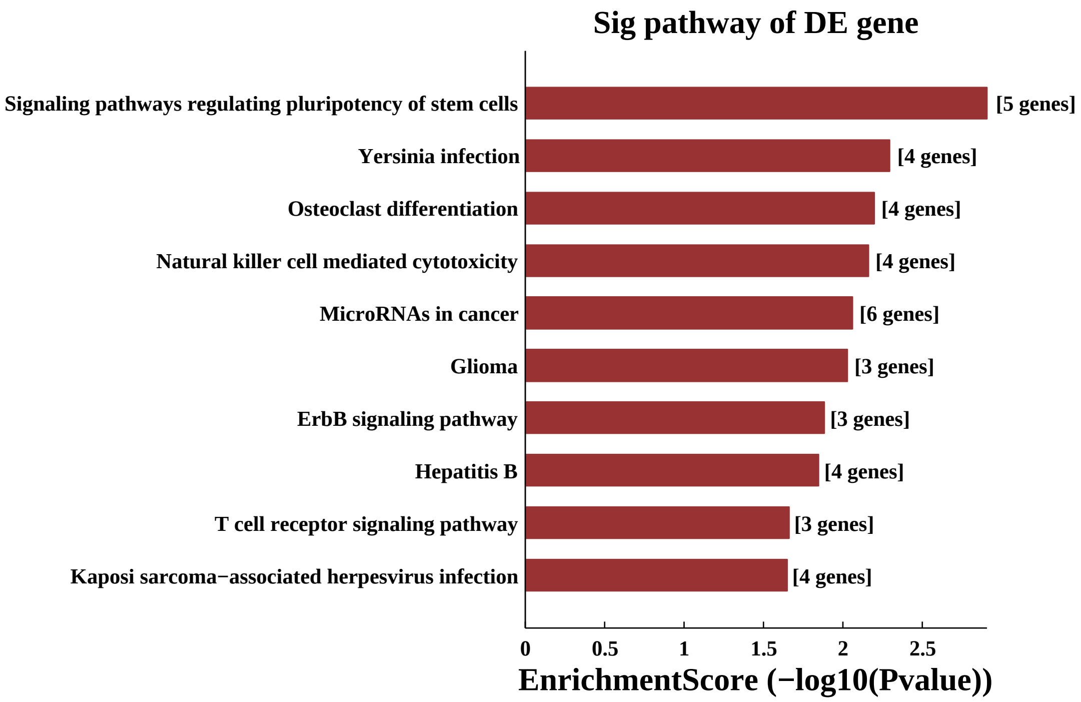
Sig pathway of DE gene
Signaling pathways regulating pluripotency of stem cells
[5 genes]
Yersinia infection
[4 genes]
Osteoclast differentiation
[4 genes]
Natural killer cell mediated cytotoxicity
[4 genes]
MicroRNAs in cancer
[6 genes]
Glioma
[3 genes]
ErbB signaling pathway
[3 genes]
Hepatitis B
[4 genes]
T cell receptor signaling pathway
[3 genes]
Kaposi sarcoma−associated herpesvirus infection
[4 genes]
0
0.5
1
1.5
2
2.5
EnrichmentScore (−log10(Pvalue))
